# Invade or die: behaviours and biochemical mechanisms that drive skin penetration in *Strongyloides* and other skin-penetrating nematodes

**DOI:** 10.1098/rstb.2022.0434

**Published:** 2024-01-15

**Authors:** Courtney R. McClure, Ruhi Patel, Elissa A. Hallem

**Affiliations:** ^1^ Molecular Toxicology Interdepartmental PhD Program, University of California, Los Angeles, CA 90095, USA; ^2^ Department of Microbiology, Immunology, and Molecular Genetics, University of California, Los Angeles, CA 90095, USA; ^3^ Molecular Biology Institute, University of California, Los Angeles, CA 90095, USA

**Keywords:** *Strongyloides*, skin-penetrating nematode, skin penetration, sensory behaviour, hookworms, astacins

## Abstract

Skin-penetrating nematodes, including the human threadworm *Strongyloides stercoralis* and hookworms in the genera *Necator* and *Ancylostoma*, are gastrointestinal parasites that are a major cause of neglected tropical disease in low-resource settings worldwide. These parasites infect hosts as soil-dwelling infective larvae that navigate towards hosts using host-emitted sensory cues such as odorants and body heat. Upon host contact, they invade the host by penetrating through the skin. The process of skin penetration is critical for successful parasitism but remains poorly understood and understudied. Here, we review current knowledge of skin-penetration behaviour and its underlying mechanisms in the human parasite *S. stercoralis*, the closely related rat parasite *Strongyloides ratti,* and other skin-penetrating nematodes such as hookworms. We also highlight important directions for future investigations into this underexplored process and discuss how recent advances in molecular genetic and genomic tools for *Strongyloides* species will enable mechanistic investigations of skin penetration and other essential parasitic behaviours in future studies.

This article is part of the Theo Murphy meeting issue ‘*Strongyloides*: omics to worm-free populations’.

## 1. Introduction

Skin-penetrating parasitic nematodes, including the human-parasitic threadworm *Strongyloides stercoralis* and hookworms in the genera *Necator* and *Ancylostoma*, are estimated to infect nearly one billion people and are a major cause of human morbidity worldwide [1–3]. Globally, *S. stercoralis—*the causative agent of strongyloidiasis—infects an estimated 600 million people [4]. Infections occur primarily when the soil-dwelling infective larvae penetrate through exposed skin; typically, infective larvae breach the skin of the feet when people walk barefoot in soil contaminated with nematode-infested faeces [5]. While many cases of strongyloidiasis are asymptomatic, acute infection with *S. stercoralis* can cause a broad spectrum of clinical symptoms that include fever, gastrointestinal pain, anorexia, diarrhoea, fatigue and respiratory distress [5]. *Strongyloides stercoralis* can also cause chronic infection owing to its ability to cycle through multiple generations within the same host, a process called autoinfection [5]. Upon subsequent immunosuppression, chronic infections can develop into hyperinfection syndrome and disseminated disease, which is often fatal [6]. Although strongyloidiasis is considered a neglected tropical disease, it has also been described as a disease of disadvantage owing to its prevalence in low-resource communities with poor sanitation infrastructure [7]. Ivermectin and albendazole are generally effective for the treatment of strongyloidiasis [8–11]; however, these drugs do not prevent reinfection and repeated treatments are often required to eliminate the infection [12]. Moreover, drug resistance is already a major problem for the treatment of *Strongyloides*-infected livestock and is likely to soon become a problem for the treatment of human strongyloidiasis [7,13,14].

Hookworms infect approximately 500 million individuals worldwide [15]. The primary species of hookworms that infect humans are *Necator americanus*, *Ancylostoma duodenale* and *Ancylostoma ceylanicum* [16–18]. Like *S. stercoralis* infections, hookworm infections are detected most frequently in socioeconomically disadvantaged communities [15]. Heavy infections with hookworms are associated with diarrhoea, abdominal pain and anemia [19]. It is estimated that approximately 4.1 million disability-adjusted life years are lost globally owing to morbidities associated with hookworm infections [15–19]. Mebendazole and albendazole are generally used to treat hookworm infections, although as with the treatment of *S. stercoralis*, the drugs are not always effective at eliminating the infection and do not prevent reinfection [15]. The potential for the emergence of drug resistance is also a concern for the treatment of hookworm infections [15].

Skin penetration, the process whereby infective larvae penetrate directly into host skin, is an essential but poorly understood step of the parasite–host interaction. This step represents a promising target for intervention, as the development of topical compounds that block skin penetration could have broad implications for nematode control. Here, we review our current knowledge of skin-penetration behaviour in *S. stercoralis,* other *Strongyloides* species*,* and hookworms. We also highlight critical areas for future investigations of skin penetration and discuss how the recently expanded genetic toolkit for *S. stercoralis* could be used to unveil the neural and molecular mechanisms that drive this behaviour.

## 2. The life cycle of skin-penetrating nematodes

**Figure 1.**
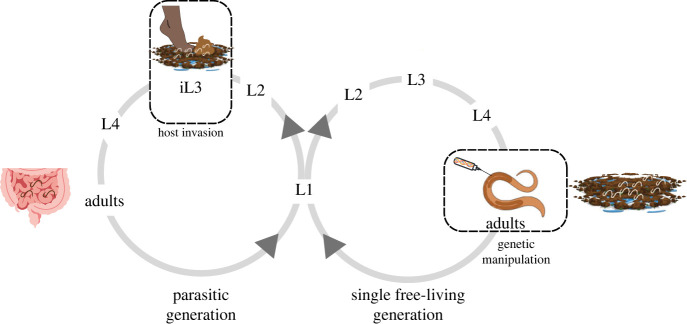
The life cycle of *Strongyloides stercoralis*. Parasitic adults reside and reproduce in the host small intestine. Their progeny exit the host in faeces as L1 larvae and then undergo either homogonic (direct) or heterogonic (indirect) development [20]. L1 larvae following the homogonic path develop through two larval stages before developmentally arresting as third-stage infective larvae (iL3s). The iL3s host seek and then invade hosts by skin penetration [21]. Inside the host, the iL3s resume development and molt twice to become parasitic adults. L1 larvae following the heterogonic path molt four times to become free-living adults. The free-living adults are amenable to genetic manipulation by intragonadal microinjection [22,23]. All progeny of the free-living adults develop into iL3s. *Strongyloides stercoralis* can also undergo an autoinfective cycle, in which the nematodes complete their life cycle inside the same host (not shown) [20]. The figure was generated using Canva, BioRender and Adobe Illustrator.

The *S. stercoralis* life cycle includes both free-living and parasitic generations [20] (figure 1). *Strongyloides stercoralis* first-stage larvae are expelled from the host in faeces. They then molt twice to form infective third-stage larvae (iL3s). The iL3s are developmentally arrested, non-feeding larvae that must invade a host to complete their life cycle [24]. The iL3s actively search for hosts using host-emitted sensory cues, including a wide array of host-emitted odorants, tastants such as sodium chloride and blood serum, and thermosensory cues such as body heat [22,25–32]. After locating a host, iL3s penetrate the skin and enter the body. Once inside the host, iL3s resume development and navigate through the host body, ultimately residing as parasitic adults in the small intestine. The parasitic adults reproduce in the small intestine, and their progeny follow one of three developmental routes: (i) larvae can develop on faeces directly into iL3s, which then find and infect a new host (i.e. homogonic development); (ii) larvae can develop into free-living adults, which sexually reproduce to generate progeny that develop exclusively into iL3s (i.e. heterogonic development); or (iii) larvae can develop into autoinfective larvae (aL3) within the host intestinal tract and then complete their life cycle in the same host. Because it can cycle through a free-living generation, *S. stercoralis* is readily amenable to molecular genetic manipulation; exogenous nucleic acids or protein can be introduced into the gonads of free-living adults to generate transgenic or mutant progeny [33]. Thus, *S. stercoralis* has become a powerful genetic model system for mechanistic studies of skin-penetrating nematodes.

The life cycle of hookworms resembles the homogonic cycle of *Strongyloides* spp. Hookworm eggs are expelled from the host in faeces into the local environment [34]. The eggs hatch into first-stage larvae; the larvae then molt twice to become iL3s. Like *Strongyloides* iL3s, hookworm iL3s are developmentally arrested and non-feeding [24]. In addition, hookworm iL3s are encased in a thick, protective exoskeleton called the sheath [18]. The iL3s stay in a state of developmental arrest until they locate and invade a host. The mechanism of entry into the host body depends on the species of hookworm: *N. americanus* are obligate skin-penetrators, whereas *Ancylostoma* spp. can enter either by skin penetration or by ingestion [35]. Development of iL3s resumes within the host; therein, larvae molt twice to become adults, which feed on intestinal blood and reproduce in the intestinal tract [15]. Unlike *Strongyloides* species, hookworms cannot cycle through a free-living generation and as a result are not yet amenable to genetic manipulation.

## 3. The structure of mammalian skin

**Figure 2.**
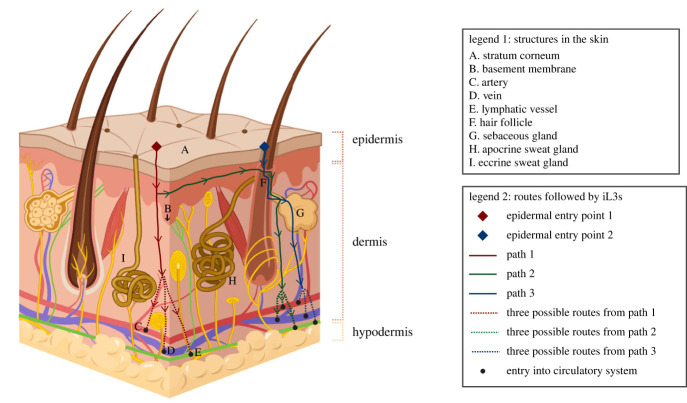
The structure of mammalian skin and potential routes of skin invasion used by hookworms. Mammalian skin or cutaneous tissue is composed of the epidermis, the dermis and various appendages [36–38]. The epidermis is avascular and is composed primarily of keratinocytes, which are stacked in multiple layers [36–39]. The outermost layer of the epidermis, termed the stratum corneum, is formed by dead keratinocytes. The epidermis and the dermis are separated via a protein-rich basement membrane [40]. The dermis is vascularized and fibrous, and is the place of origin of several skin appendages, including hair follicles and sweat glands [38]. The cutaneous tissue lies on top of the subcutaneous tissue or hypodermis; the hypodermis is composed of fat and traversed by circulatory vessels [41,42]. The structures in the skin that are relevant to skin penetration are labelled A-I and listed in legend 1. Hookworm iL3s follow different routes to travel into and through the skin. As indicated in legend 2, the diamonds depict two possible points of entry that iL3s might use to invade the epidermis, lines depict distinct paths of migration of iL3s through the tissue, arrows indicate the direction of migration of iL3s, and circles depict points where iL3s might enter the circulatory fluid. Whether *Strongyloides* spp. follow similar migratory routes during skin penetration remains to be determined. The figure was generated using BioRender and Adobe Illustrator.

Upon host contact, skin-penetrating iL3s encounter the first barrier to infection: the skin. Mammalian skin is composed of two major layers: the epidermis and the dermis (figure 2) [36–38]. The epidermis, which is the outermost layer of skin, is avascular and composed primarily of layers of keratinocytes, or epidermal skin cells [36–39]. The outermost layer of the epidermis, termed the stratum corneum, is formed by dead keratinocytes [36–39]. An extracellular matrix called the basement membrane separates the epidermis from the underlying dermis [40]. The dermis contains a meshwork of collagen and elastic fibres that are embedded in a gel-like, glycosaminoglycan-rich material called the ground substance [38]. The dermis also contains cells such as fibroblasts and macrophages, and it is traversed by both blood and lymphatic vessels [38]. In addition, hair follicles and sweat glands originate within the dermis and extend upwards through the epidermis to the skin surface (figure 2) [36]. Hair follicles are associated with sebaceous glands; these glands secrete lipids that moisturize the skin surface. The cutaneous tissue (i.e. the epidermis and the dermis) is followed by the subcutaneous tissue, or hypodermis. The hypodermis is composed of fat cells, blood vessels, and collagen [41,42]. Thus, mammalian skin is an intricate, multi-layered organ that iL3s must navigate through to establish an infection in a host.

## 4. Skin-penetration behaviours of *Strongyloides* species

The skin-penetration behaviour of *S. stercoralis* and other *Strongyloides* species is poorly understood. Early *ex vivo* studies revealed that *S. ratti* iL3s penetrate shaved rat skin isolated from young adult rats within 1 min of placement on the skin [43]. After 3 min, iL3s can be found in the dermis, indicating that they travelled through the epidermis and penetrated through the basement membrane into the dermis [43]. By contrast, *S. ratti* iL3s penetrate the skin of older rats more slowly, with the first iL3s penetrating the skin within 2–3 min; the slower rate of skin penetration in older rats may reflect the increase in polymerization of the basement membrane and ground substance that occurs with age [43]. *Strongyloides ratti* iL3s are capable of penetrating either directly through the stratum corneum or through hair follicles [44–46]. Although the behavioural sequences that lead to skin penetration have not been studied, *Strongyloides* iL3s are known to penetrate head-first [45]. Inside host skin, *S. stercoralis* iL3s migrate through the skin at rates of up to 5–15 cm h^−1^ [47]. Skin-penetration assays using iL3s of the rat parasite *Strongyloides venezuelensis* that were extracted from a host revealed that iL3s rapidly lose the ability to penetrate after they have entered the host [48]. Although *Strongyloides* iL3s do not have a traditional sheath like hookworm iL3s, *S. ratti* iL3s have a proteinaceous surface coat surrounding their epicuticle that is derived at least in part from excretory-secretory (ES) products [49,50]. This surface coat, which is shed during skin penetration, may serve as a protective layer for iL3s in the environment [49,50].

Skin-penetrating nematodes generally establish an infection in only a few host species. For example, *S. ratti* infects rats, while *S. stercoralis* infects humans, non-human primates, dogs, and cats [51–56]. However, *Strongyloides* iL3s can penetrate the outer skin layer of some non-host species, where they can cause a condition known as larva currens, which is characterized by a pruritic rash that follows the trail of migrating larvae [57–60]. In the case of the rat parasite *S. ratti*, a comparison of skin-penetration frequencies on skin from different species in an *ex vivo* assay revealed that iL3s penetrate host skin at a higher frequency than skin from non-host species such as cats, dogs, and birds [61]. This finding raises the possibility that iL3s use host-specific sensory cues to distinguish host from non-host skin. However, the nature and identity of these cues remains unclear. It is also possible that differences in rates of skin penetration on host versus non-host species reflect species-specific differences in the mechanical properties of the skin. Such differences might include variations in the density of hair follicles as well as variations in the thickness of either the epidermis or dermis.

## 5. Skin-penetration behaviours of hookworms

The behaviours of hookworm iL3s upon contact with the surface of skin are largely unknown, although some details have emerged from *ex vivo* studies of *N. americanus* on rodent skin and the cat parasite *Ancylostoma tubaeforme* on cat skin [46,62]. Like *Strongyloides* iL3s, hookworm iL3s were found to penetrate head-first [46]. In the case of *N. americanus*, the iL3s penetrated the skin while molting out of their sheath [46]. Following skin penetration by *N. americanus*, large numbers of sheaths were detected on the skin surface, while larvae were found within the epidermis. This suggests that most *N. americanus* iL3s exsheath just prior to or during epidermal invasion [63]. By contrast, some *A. tubaeforme* iL3s appear to retain their sheath during skin invasion [62].

The route of skin penetration (i.e. initial entry into the epidermis, invasion of the dermis and hypodermis, and ultimately, access to the circulatory fluid) was determined by placing hookworm iL3s on mammalian skin, followed by time-lapse serial sectioning and electron microscopy [62,64–66]. The route of migration through the skin was deduced by detection of larvae themselves or tracks of larvae in the tissue. The iL3s enter the epidermis either through fissures between keratinocytes in the stratum corneum (figure 2, epidermal entry point 1) or via hair follicles (figure 2, epidermal entry point 2) [62,64]. Upon entering the epidermis, iL3s either directly migrate from the epidermis to the dermis (figure 2, path 1) or move parallel to the surface of the skin (figure 2, path 2) [64]. An alternate path of migration into the dermis involves the hair follicle system: entry into a hair follicle may occur either from the surface of the epidermis (figure 2, path 3), as stated above, or during lateral movement within the epidermis (figure 2, path 2); thereafter, the iL3s might exit the hair follicle into the dermis via a sebaceous gland (figure 2, paths 2 and 3) [66]. Possible points of access to the circulatory system include arteries, veins and lymphatic vessels in both the dermis and hypodermis (figure 2, paths 1–3) [66]. In addition, the preferred route of skin invasion varies depending upon the part of the body that was first encountered by the worm. For example, in the case of the cat and dog hookworm *Ancylostoma braziliense*, iL3s that invaded the metacarpal footpads of puppies, which are devoid of hair follicles and apocrine sweat glands, either stayed in the epidermis or migrated laterally into portions of the feet that have hair follicles [65]. In the hairy portions of the feet, larvae were detectable in hair follicles, apocrine sweat glands, sebaceous glands and the dermis [65]. Importantly, all the above-mentioned studies relied on endpoint observations of the locations of either larvae or tracks in the different layers and appendages of the skin to infer the route of skin penetration. In the future, real-time observations of individual larvae, in combination with definitive markers for structures such as hair follicles, sweat glands and sebaceous glands will enable determination of the preferred route of skin invasion.

The kinetics of skin penetration vary widely depending on the species of hookworms and the part of the body to which the iL3s are initially exposed. Both *N. americanus* and *A. braziliense* enter human and dog skin at roughly similar rates—roughly 20–30% of the infective dose was detectable in the skin within 30 min of exposure [66,67]. By contrast, less than 10% of the infective dose of *A. ceylanicum* and *Ancylostoma caninum* iL3s were detectable in the skin at the same time-point [64]. Additionally, *A. braziliense* iL3s took longer to invade the metacarpal foot pads of dogs than the lateral surfaces, perhaps because the surfaces of the metacarpal foot pads are tougher and devoid of hair follicles [65].

## 6. The role of sensory cues in stimulating skin penetration

Skin-penetrating iL3s are robustly attracted to host body heat and will engage in long-range migration up a thermal gradient [22,28,29,32,68–73]. In addition, iL3s are more active at host body temperature than room temperature, which may allow iL3s to rapidly explore the skin surface and find an optimal spot for entry [25,28,67,74]. Skin-penetrating iL3s also engage in host seeking and environmental navigation in response to a wide array of host-emitted olfactory and gustatory cues (e.g. skin and sweat odorants, sodium chloride, serum and sweat), as well as cues emitted by host-associated and environmental bacteria [21,22,25–27,30,31,35,72,75–86]. The role of sensory cues in mediating skin penetration by iL3s remains poorly understood. In the case of *S. ratti*, host body temperature stimulates increased skin penetration—in an *ex vivo* assay, a higher percentage of *S. ratti* iL3s penetrated rat skin at 37°C than 20°C [61]. In the case of hookworms, skin lipids stimulate skin penetration [35,67]. For example, in an *ex vivo* assay with *N. americanus* iL3s on human skin, removal of skin surface lipids significantly reduced the proportion of iL3s that penetrated the skin by about 4-fold [67]. This behavioural phenotype was partially rescued by reintroducing the lipids onto the skin surface [67]. The role of other sensory cues in stimulating skin penetration has yet to be investigated.

## 7. The role of metalloproteases during skin penetration

The process of invading host skin involves both specific behaviours that drive the iL3s head-first into the skin and the secretion of enzymatic agents that enable skin invasion by digesting the skin. Early studies of both *Strongyloides* spp. and hookworms provided several lines of evidence demonstrating that ES products produced by iL3s during skin penetration partially degrade the skin to enable larval entry [50,67,87–89]. For example, studies of *N. americanus* detected epidermal damage up to 100 µm away from the anterior end of an invading iL3 [67].

The oesophageal glands of *N. americanus* iL3s prior to skin penetration have more secretory granules and are about 1.5-fold larger in diameter than those of iL3s that have completed skin penetration [90]. Moreover, small granules were detectable in the oesophageal lumen of *N. americanus* iL3s that had penetrated skin, whereas none were detectable prior to penetration [90]. In *in vitro* assays, *N. americanus* iL3s hydrolyse the substrate azocoll, an insoluble collagen with an attached dye that is commonly used to detect protease activity [91], suggesting that the parasite secretes proteolytic enzymes [67]. Intriguingly, the hydrolysis of azocoll occurred most efficiently in the temperature range of approximately 30–40°C and the pH range of approximately 5–8 [67]; the temperature of human skin measures between 30 and 35°C and the pH of the human epidermis is 4.1–5.8 [92,93]. Taken together, these data suggest that skin-penetrating iL3s secrete proteolytic enzymes that are highly active in the microenvironment of the skin and help to break down the skin, aiding in the process of skin penetration.

Subsequent studies of the ES products of skin-penetrating iL3s provided evidence that the active components of ES products that digest skin are zinc-dependent metalloproteases. ES products from *S. stercoralis* iL3s were found to contain metalloprotease activity and exposing worms to the zinc metalloprotease inhibitor 1,10-phenanthroline greatly reduced the rate of skin penetration in an *ex vivo* assay with excised rat skin [87]. Partial purification of the proteases in ES products led to the identification of an approximately 40 kDa protein [87]; this protein was subsequently termed strongylastacin and identified as a member of the astacin family of zinc metalloproteases [94]. Active fractions containing strongylastacin were capable of degrading both elastin and azocoll [87]. Similarly, an astacin metalloprotease called MTP-1 was found to be secreted by hookworm iL3s [95,96]. *Ancylostoma caninum* MTP-1 is produced in the secretory granules of the oesophagus, is capable of digesting connective tissue, and like strongylastacin, is inhibited by the zinc chelator 1,10-phenanthroline [97]. Moreover, incubating *A. caninum* iL3s in anti-MTP-1 serum inhibited skin penetration in an *ex vivo* assay with dog skin [97]. Together, these studies provide strong evidence that astacin metalloproteases play an important role in digesting skin during penetration. Interestingly, both strongylastacin and MTP-1 are immunogenic and have been proposed as possible vaccine candidates [98–100].

**Figure 3.**
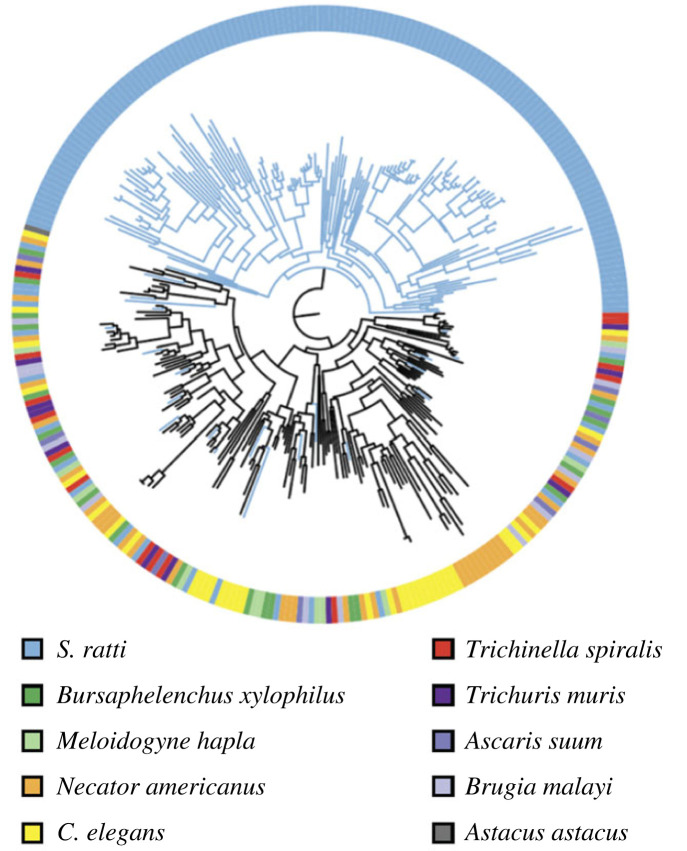
The astacin gene family is highly expanded in *Strongyloides* species. A phylogenetic dendrogram of the astacin genes of selected nematode species. *Strongyloides ratti* contains a large expansion of the astacin gene family. The figure is reprinted from Hunt *et al.* [101].

More recent genomic analyses revealed that the *S. stercoralis* genome contains 237 astacin genes, while the *S. ratti* genome contains 184 astacin genes [101,102]. The astacin gene family is highly expanded in *Strongyloides* species relative to other nematode species [101–103] (figure 3). For example, the genome of the free-living nematode *Caenorhabditis elegans* contains only 40 astacin genes [102,104]. In *C. elegans*, astacins play a role in cuticle formation, molting and egg hatching [102,105]. While close homologues of the *C. elegans* astacins may play similar roles in *Strongyloides*, many of the *Strongyloides* astacin genes, including strongylastacin, are part of a parasite-specific expansion of the gene family, leading to the hypothesis that astacins play a critical role in parasite-specific behaviours such as skin penetration and intra-host migration [101,102]. Moreover, a subset of the parasite-specific astacin genes are highly upregulated in iL3s relative to other life stages [101] and highly enriched in the iL3 secretome [106,107]. Based on these findings, astacins that are upregulated in iL3s are thought to play a role in skin penetration and tissue migration [103,106]. However, there is still no direct evidence for the role of astacins in either of these processes.

## 8. *Strongyloides* spp. are genetically tractable models for the study of skin penetration

Significant advances over the past two decades have situated *Strongyloides* spp. as model parasitic nematodes that are ripe for genetic, neuronal and genomic exploration. Notably, in the past few years there has been a rapid expansion in the number and diversity of tools that can be used to characterize and interfere with *Strongyloides* genetic pathways and neuronal activity. These advances will facilitate a mechanistic understanding of the biology and behaviour of skin-penetrating nematodes, which were previously not amenable to genetic manipulation. The single free-living generation of *Strongyloides* spp. is the key factor that has enhanced the genetic tractability of this species relative to other parasitic nematode species.

Transgenesis is routinely achieved by intragonadal microinjection of transgene-encoding DNA into *Strongyloides* free-living adults [108–112]. The design of constructs for transgenesis is bolstered by the availability of the fully sequenced genomes of both *S. stercoralis* and *S. ratti*, which can be accessed in WormBase ParaSite, a web-based portal [101,113,114]. In addition, transgenesis-based tools to study neuronal function, including chemogenetic neuronal silencing [29,115] and functional imaging using fluorescent biosensors [29], have been recently optimized for use in *Strongyloides* spp., which allows for mechanistic exploration of the neuronal basis of parasitic behaviour [29]. The growing genetic toolkit for *Strongyloides* spp. now gives us a unique opportunity to uncover the genetic and neural mechanisms that underlie skin penetration [26,28,29,116].

Generation of transgenic *Strongyloides* iL3s is accomplished by microinjection of transgene-encoding DNA into the gonads of free-living females or males using techniques adopted from *C. elegans* [108–110,112]. Some important considerations for the design of DNA constructs for expression in *Strongyloides* are as follows: (i) inclusion of a 3′ untranslated region (3′ UTR) from the *Strongyloides* genome, such as the *Ss-era-1* 3′ UTR, improves the efficiency of transgenesis [117]; (ii) codon optimization improves transgene expression [118]; and (iii) gene annotations in WormBase ParaSite should be carefully examined for accuracy given that errors in automated gene annotations are common and require manual correction using publicly available RNA-sequencing (RNA-seq) data [29,119]. The injected DNA forms extrachromosomal arrays and the expression of transgenes from these arrays can be detected in the F_1_ generation either by direct visualization or reverse transcription-polymerase chain reaction [108–110,117]. However, transgene expression is no longer detectable from the F_2_ generation onwards, probably owing to silencing of the extrachromosomal arrays [117]. One solution to this problem is to force chromosomal integration of foreign DNA via transposon-mediated integration methods such as the *piggyBac* system [120].

Techniques for targeted gene disruption or silencing that have been developed for *Strongyloides* spp. include CRISPR/Cas9-mediated targeted mutagenesis and RNA interference (RNAi) [23,121,122]. Gonadal microinjection of either plasmids that encode a single guide RNA and Cas9 protein or CRISPR/Cas9 ribonucleoprotein complexes resulted in heritable genetic mutations [122]. The efficiency of gene targeting at the *S. stercoralis unc-22* locus, which encodes a twitchin protein [123,124], was at least 20–30% based on phenotypic analysis of the F_1_ larvae [122]. Homology-directed repair (HDR) can also be exploited in *Strongyloides* spp. to insert a DNA cassette at a locus of interest by injecting a repair template with the appropriate homology arms along with the rest of the CRISPR/Cas9 machinery. One advantage of the HDR-based approach is that mutant larvae can be easily identified by using a repair template that encodes a fluorophore such as green fluorescent protein or mScarlet [122]. CRISPR has now been used successfully to disrupt several *S. stercoralis* genes [23,26,28,29,122,125–127]. For example, the cGMP-gated cation channel subunit gene *tax-4* was shown to be required for both the odour-driven and temperature-driven host-seeking behaviours of *S. stercoralis* iL3s using a targeted mutagenesis approach [26,28,29,122]. A distinct method that has been used successfully to silence genes in *S. ratti* is RNAi [121]. Incubation of *S. ratti* iL3s with small interfering RNAs (siRNAs) complementary to the *daf-12* gene resulted in a reduction of *daf-12* expression by 3-fold within 48 h of incubation [121]. In these experiments, the proportion of post-parasitic *S. ratti* L1 larvae that underwent direct development to the iL3 stage after soaking with *daf-12* siRNAs was 6-fold lower than mock-treated controls [121]. The ability to investigate specific genes with these reverse genetic approaches will allow for the study of the molecular mechanisms that underlie skin penetration in *Strongyloides* spp.

Approaches for analysis of neuronal function and activity have also been successfully applied to *Strongyloides* spp. [29]. In a recent study, neuronal silencing was achieved by driving expression of the histamine-gated chloride channel HisCl1 in neurons of interest followed by exposure of the worms to exogenous histamine [29]. Using this technique, the AFD neurons of *S. stercoralis* were shown to be required for heat seeking. Moreover, functional imaging with the ratiometric calcium indicator yellow cameleon YC3.60 [116] and the cGMP sensor FlincG3 [128] revealed that the *S. stercoralis* AFD thermosensory neurons display parasite-specific adaptations in their response properties that support long-range navigation towards human hosts [29]. Similar approaches can now be used to identify and functionally characterize sensory neurons required for skin penetration.

Efforts to further expand the functional genomics toolkit of *Strongyloides* could include single-cell RNA-sequencing (scRNA-seq), which has been applied to *C. elegans* [129]. The application of scRNA-seq in *Strongyloides* spp. would provide an unbiased approach to the identification of genes that drive parasite-specific behaviours such as skin penetration. In addition, the auxin-inducible degradation system, which has been used successfully in *C. elegans* [130], could be used for conditional protein degradation in *Strongyloides*. Further technical developments in these areas could greatly facilitate the study of skin penetration and other behaviours that enable skin-penetrating nematodes to locate, invade, and establish an infection in human hosts.

## 9. Conclusion and future directions

Our understanding of skin penetration and its underlying mechanisms remains limited. Given the recent advances in tool development for *S. stercoralis* and *S. ratti,* studies of the molecular, neural and behavioural bases of skin penetration will be increasingly feasible in the coming years. In addition, important areas for future research include investigations into the roles of mechanosensation, gustation, olfaction and other sensory modalities in stimulating skin-penetration behaviour. Infections with skin-penetrating nematodes remain a major health concern, particularly in low-resource, marginalized communities worldwide. A better understanding of skin penetration may lead to the development of novel topical anthelmintics that block this process and thereby prevent harmful nematode infections.

## Data Availability

This article has no additional data.
